# Considerations for the Feasibility of Neutralizing Antibodies as a Surrogate Endpoint for COVID-19 Vaccines

**DOI:** 10.3389/fimmu.2022.814365

**Published:** 2022-04-27

**Authors:** Jianyang Liu, Qunying Mao, Xing Wu, Qian He, Lianlian Bian, Yu Bai, Zhongfang Wang, Qian Wang, Jialu Zhang, Zhenglun Liang, Miao Xu

**Affiliations:** ^1^ National Institutes for Food and Drug Control, Beijing, China; ^2^ NHC Key Laboratory of Research on Quality and Standardization of Biotech Products, Beijing, China; ^3^ NMPA Key Laboratory for Quality Research and Evaluation of Biological Products, Beijing, China; ^4^ Guangzhou Laboratory, Guangzhou, China

**Keywords:** COVID-19 Vaccines, surrogate endpoints, neutralizing antibody, standard neutralization test assay, national standard

## Abstract

To effectively control and prevent the pandemic of coronavirus disease 2019 (COVID-19), suitable vaccines have been researched and developed rapidly. Currently, 31 COVID-19 vaccines have been approved for emergency use or authorized for conditional marketing, with more than 9.3 billion doses of vaccines being administered globally. However, the continuous emergence of variants with high transmissibility and an ability to escape the immune responses elicited by vaccines poses severe challenges to the effectiveness of approved vaccines. Hundreds of new COVID-19 vaccines based on different technology platforms are in need of a quick evaluation for their efficiencies. Selection and enrollment of a suitable sample of population for conducting these clinical trials is often challenging because the pandemic so widespread and also due to large scale vaccination. To overcome these hurdles, methods of evaluation of vaccine efficiency based on establishment of surrogate endpoints could expedite the further research and development of vaccines. In this review, we have summarized the studies on neutralizing antibody responses and effectiveness of the various COVID-19 vaccines. Using this data we have analyzed the feasibility of establishing surrogate endpoints for evaluating the efficacy of vaccines based on neutralizing antibody titers. The considerations discussed here open up new avenues for devising novel approaches and strategies for the research and develop as well as application of COVID-19 vaccines.

## Introduction

The current coronavirus disease 2019 (COVID-19) pandemic caused by the severe acute respiratory syndrome coronavirus 2 (SARS−CoV−2) is a disaster of unprecedented magnitude in modern times. On the other hand, the rapid research and development (R&D) and application of COVID-19 vaccines in response to the pandemic can be regarded as a miracle in the history of vaccine development. Over a span of less than 2 years, a total of 31 different types of COVID-19 vaccines around the world have been granted Emergency Use Authorizations (EUAs) or marketing approvals (1–3). As of January 15, 2022, more than 9.3 billion doses of COVID-19 vaccines have been administered worldwide (4), but vaccination rates remain low in many regions and countries. The current lots of vaccines are far from perfect. Reduction in immunity over a period of time and lower efficiency against variants seem to be the major concerns. However, vaccines do offer a means to combat the pandemic. The R&D and application of vaccines with superior immunogenicity and universal effectiveness against all SARS−CoV−2 variants, are of utmost priority in addition to increasing vaccination coverage and administrating of a third (booster) COVID-19 vaccine dose (5–7).

Phase III clinical trials are the main rate-limiting steps in vaccine R&D and application. In countries with high COVID-19 vaccination coverage or viral prevalence, it is difficult to conduct trials on the clinical efficacy of vaccines. The search for methods to rapidly and effectively evaluate vaccine’s effectiveness has, therefore, become a bottleneck in subsequent vaccine R&D efforts. Surrogate endpoints may effectively save clinical time and are compliant with ethical standards. There has been a successful history of using antibodies as surrogate endpoints for other licensed viral vaccines. The key to establishing surrogate endpoints relies on finding a correlationship between vaccine-induced immune responses and the level of protection (8). The cellular and humoral immunity induced by the vaccine synergistically protects the human body from viral infection (9, 10). Antibodies, especially neutralizing antibodies, are key immunological markers that signal the elicitation of defense responses for the prevention and control of viral infections and disease onset. Consequently, the respective protective antibody levels have been used as surrogate endpoints for many viral vaccines such as influenza virus vaccine, measles vaccine, Japanese encephalitis vaccine, rabies vaccine, polio virus vaccine, hepatitis A vaccine, enterovirus 71 (EV71) vaccine, varicella vaccine and hepatitis B vaccine (**Table 1**) (11–21). In the case of measles, a pre-exposure neutralizing antibodies level in serum samples was positively correlated with clinical protection. Based on this finding, neutralizing antibodies titers of >120 mIU/mL were considered as a reliable surrogate endpoint for measles vaccine (12, 22). Similarly, a titer of ≥ 20 mIU/mL was defined as seroconversion level for hepatitis A vaccine. This seroconversion rate showed a high level of agreement with clinical efficacy data of hepatitis A vaccine. Subsequently, in 2012, hepatitis A virus (HAV) IgG ≥ 20 mIU/mL was included as the antibody threshold level for clinical effectiveness in the World Health Organization (WHO) technical reports on hepatitis A vaccines (17, 23–25). A neutralizing antibody titer of 1:32 for EV71 vaccine has been recommended as the immunological surrogate endpoint because of its association with protection against EV71 (18, 19). Previous clinical studies have confirmed that COVID-19 vaccine-induced humoral immunity generates effective neutralization antibodies against SARS-CoV-2 (26, 27). The studies by Khoury et al. and Earle et al. demonstrated that there is a correlation between the level of neutralizing antibodies responses to SARS-CoV-2 and the protection level of the vaccine, which raises the possibility for the establishment of surrogate endpoint (28, 29). T cell response is essential in inducing high-affinity antibodies and immune memory (30). SARS-CoV-2 specific responsive T cell numbers are associated with protection against COVID-19 and accelerated viral clearance (31, 32). In fact, mRNA vaccination induced early CD4^+^ T cell responses have been shown to correlate well with long-term humoral immunity. Robust cellular immune memory to SARS-CoV-2 and its variants persist for at least 6 months after mRNA vaccination (33). However, the correlation between the T cell immunity and the protection level of COVID-19 vaccines is not very clear. In addition, compared to T cell immunity, antibody threshold levels are commonly used as surrogate endpoints for viral vaccines due to the ease of establishment of standardized test methods.

**Table 1:** Recommended immunological surrogate endpoints for licensed viral vaccines.

Vaccine name	Vaccine type	Route of virus transmission	Recommended clinical surrogate endpoint for vaccine	Ref
Influenza vaccines	Inactivated vaccines	Respiratory tract	1. Significant increase in seroconversion factor or hemagglutination inhibition (HI) antibody titer of > 40%; 2. Increase in GMT of > 2.5; 3. Proportion of subjects with HI titer ≥ 1:40 or single radial hemolysis (SRH) area > 25 mm² > 70%	(11)
	Split virus vaccines			
	Subunit vaccines			
Measles vaccines	Live attenuated vaccines	Respiratory tract	Hemagglutinin (H)- and fusion protein (F)-specific neutralizing antibody titer > 120 mIU/mL in the plaque reduction neutralization test (PRNT)	(12)
Japanese encephalitis vaccines	Live attenuated vaccines	Mosquito vectors	Baseline negative: PRNT_50_ ≥ 1:10	(13)
			Baseline positive: 4-fold increase in PRNT_50_	
Rabies vaccines	Inactivated and live attenuated vaccines	Animals	The WHO states that vaccines with a potency of 2.5 IU/dose can induce adequate immunogenicity and provide protective effects with the generation of antibody concentrations of > 0.5 IU	(14)
Polio vaccines	Inactivated and live attenuated vaccines	Gastrointestinal tract	Oral live attenuated vaccine: neutralizing antibody titer of 1:4–1:8 or 4-fold increase in antibody titer; inactivated vaccine: neutralizing antibody titer of 1:8 or 4-fold increase in antibody titer	(15, 16)
Hepatitis A vaccines	Inactivated and live attenuated vaccines	Gastrointestinal tract	Anti-hepatitis A virus (HAV) IgG ≥ 20 mIU/mL	(17)
Enterovirus 71 (EV71) vaccines	Inactivated vaccines	Gastrointestinal tract	Neutralizing antibody titer of 1:32	(18, 19)
Varicella vaccines	Live attenuated vaccines	Contact	Titer of antibodies to the varicella-zoster virus (VZV) glycoprotein measured by ELISA ≥ 5 gpELISA units	(20)
Hepatitis B vaccines	Recombinant vaccines	Blood	Percentage of subjects with hepatitis B surface antibody (anti-HB) titer ≥10 mIU/mL	(21)

At present, a large number of ongoing clinical trials of COVID-19 vaccines have not reported the threshold antibody levels of protection of their respective study populations. In addition, a lack of standardized neutralizing antibody detection methods and the effects of variants on the serum neutralizing activity of vaccines also pose challenges to the establishment of surrogate endpoints (5). This present paper provides a review of the current status of research on neutralizing antibody responses and their utility in gauging the effectiveness of the various COVID-19 vaccines. An analysis of the feasibility of establishing surrogate endpoints based on neutralizing antibody levels in the hope of opening up new horizons for the R&D of an efficient and expedited application of COVID-19 vaccines.

## Current Status of R&D and Application of COVID-19 Vaccines

**Figure 1:**
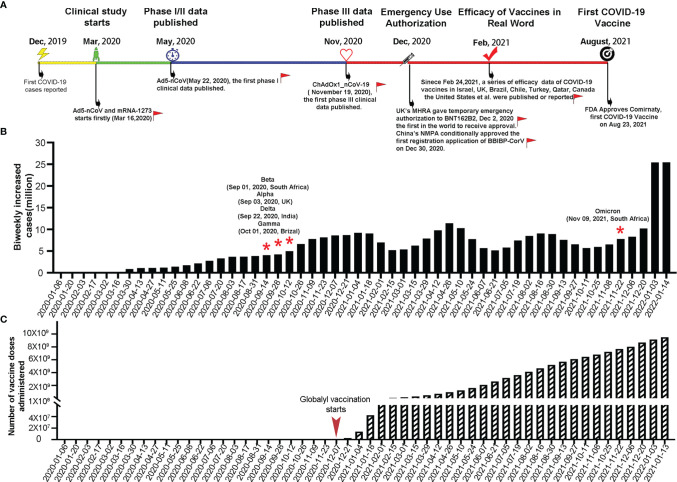
Key milestones in COVID-19 vaccine R&D, biweekly increases in COVID-19 cases worldwide, and total number of COVID-19 vaccine doses administered worldwide. **(A)** Timeline of the R&D of COVID-19 vaccines. Red flags indicate the key milestones in global COVID-19 vaccine R&D and vaccination with the corresponding dates indicated in parentheses. **(B)** Biweekly increases in COVID-19 cases worldwide with red stars denoting the time points of emergence of SARS−CoV−2 variants (data source: https://covid19.who.int/, https://cov-lineages.org/index.html). **(C)** Total number of COVID-19 vaccine doses administered worldwide (data source: https://github.com/owid/covid-19-data/blob/master/public/data/README.md).

The R&D and application of COVID-19 vaccines have progressed at an unparalleled pace in a global effort to control the ongoing pandemic. **Figure 1A** shows the major milestones in COVID-19 vaccine R&D and application. In December 2020, merely 1 year after the first report of COVID-19, the BNT162b2 vaccine jointly developed by Pfizer and BioNTech was granted the first-ever EUA in the United Kingdom (UK) (34). This marked the start of large-scale, rapid vaccinations around the world (**Figure 1C**). Currently, 31 vaccines have received EUAs or conditional marketing authorizations, including two mRNA, eleven inactivated viruses, five adenoviral vectors, twelve recombinant subunits, and one DNA vaccine (1–3). As of January 15, 2022, eight COVID-19 vaccines have been approved for emergency use by the World Health Organization; namely, BNT162b2, AZD1222, Ad26.COV2.S, mRNA-1273, BBIBP-CorV, Coronavac, NVX-CoV2373 and Covaxin (35, 36).

At present, the percentage of fully vaccinated people who have received all recommended doses of a COVID-19 vaccine has exceeded 50% in the United States of America (USA), Canada, and many developed countries of the European Union. In the two most populous countries in the world (China and India), the number of COVID-19 vaccine doses administered has reached 2.93 billion and 1.56 billion, respectively, which jointly account for approximately half of the total doses administered globally (37, 38). With rapid vaccination efforts, the number of new COVID-19 cases worldwide showed a significant decrease between January and March 2021 (**Figure 1B**). However, the emergence of the Alpha, Beta, Gamma, Delta and Omicron variants with high transmissibility and an immune evasion ability has resulted in new waves of the pandemic, with the fourth wave caused by the Omicron variant on the rapid upswing (**Figure 1B**). In particular, the highly divergent Omicron variant, carrying over 30 mutations in the spike protein, has a substantial growth advantage over previous variant and has been identified in 149 countries since November 2021 (39–41). Neutralization titer against Omicron is significantly reduced in convalescent sera from previous SARS-CoV-2 patient, sera after vaccination and therapeutic monoclonal antibodies (42–44). The combined effects of the spread of the Delta and Omicron variants and decrease in neutralizing antibody titers with time after vaccination, have led to an increase in breakthrough infection rates in the real world. Therefore, current vaccines are no longer capable of effectively preventing breakthrough infections and the transmission of variants. The goal of vaccination seems to have shifted from preventing disease onset to reducing the number of critically ill patients and deaths (38, 45–47).

To cope with the aforementioned situation, booster vaccination has become the strategy of choice for certain countries. Studies have indicated that a 5–25-fold increase in neutralizing antibody titer could be achieved after the administration of a third vaccine dose, which indicates a significant increase in efficacy compared with two vaccine doses (48–51). Real-world data from Israel showed that 12 days after the booster dose, the rate of confirmed infection was lower in the booster group than in the non-booster group by a factor of 11.3 (95% confidence interval (CI): 10.4 to 12.3), and the rate of severe illness decreased by a factor of 19.5 (95% CI: 12.9 to 29.5) (9). On October 21, 2021, Pfizer and BioNTech announced the first phase 3 clinical trial data of a booster dose of their COVID-19 vaccine. When Delta is the prevalent strain, the protection rate of booster vaccine is as high as 95.6% (52). Compared with prime COVID-19 vaccination, a homologous and heterologous booster dose elicits potent neutralization titers against Omicron variant, which increasing vaccine effectiveness (28, 53–57). Currently, some countries, including Israel, the USA, the UK, Switzerland, Germany, and China, have already launched booster vaccination campaigns (58–60). However, research data on booster vaccination are relatively scarce. In particular, the duration of retention of adequate neutralizing antibody levels and mechanisms of titer decline after the booster shot remain unclear. Importantly, the safety of this approach has also not been adequately demonstrated yet (61). Considering that infectious diseases know no borders and the vaccination coverage rates worldwide remain low, the WHO has repeatedly called for developed countries to refrain from the widespread rollout of booster shots until the percentage of fully vaccinated people of other nations have been adequately increased because the vaccination of a high percentage of the world’s population may serves as the most effective pandemic prevention and control strategy (62, 63). On October 26, 2021, the WHO Emergency Committee acknowledged that the COVID-19 pandemic is far from over, calling for the development of vaccines, diagnostic tools and therapeutics for long-term control of the pandemic (7). To curb the spread of variants, institutions and companies around the world have embarked on the R&D of multi-variant COVID-19 vaccines (64–66).

## Studies on the Correlation Between Neutralizing Antibody Levels and Immunological Protection

The establishment of immunological surrogate endpoints is aimed at finding relevant indicators of vaccine protection through animal or human challenge experiments, and then using clinical data to obtain the relationship between immune protection indicators and clinical protection through different data statistical analysis models. Such an exercise produces the initial surrogate endpoints. Neutralizing antibody levels are generally used as the primary surrogate endpoint of the immunological protection of viral vaccines. Results of animal challenge models, efficacy of monoclonal antibodies, data from clinical trials of different vaccines, and real-world data of COVID-19 vaccines in many countries, have demonstrated the existence of a certain correlation between neutralizing antibody levels and an vaccine’s effectiveness (5, 28, 67–71).

### Immunological Protection in Nonhuman Primates

Data from preclinical nonhuman primates challenge studies investigating the correlation between neutralizing antibody levels and vaccine effectiveness have indicated that vaccines developed using various technologies are capable of inducing the production of neutralizing antibodies in nonhuman primates. The neutralizing antibody titers induced in nonhuman primates by the vast majority of vaccine candidates tested in Phase III clinical trials are within the range of 100–5000. Despite significant differences in neutralizing antibody levels, all of these vaccines are capable of reducing the pathological response and viral load in the bronchoalveolar lavage or lungs to a certain extent (**Table 2**). Studies on the effectiveness of adenoviral vector vaccines (Johnson & Johnson) (76) and DNA vaccines (80) have revealed that the viral load in lungs is negatively correlated with the neutralizing antibody titer (R values: −0.5714–−0.7702) and that the neutralizing antibody titer must not be lower than 100–250 for the vaccine in order to provide full protection.

**Table 2:** Protective effects of neutralizing antibodies in nonhuman primates challenge studies.

Vaccines	Immunization procedure			Challenge dose	Neutralizing antibodies		Viral load (copies/ml) in BAL fluid		Ref
	Dosage	Doses	Interval (weeks)		Method	geometric mean titer(GMT)	Control	Vaccine	
mRNA-1273	100 μg	2	4	7.6 × 10^5^ PFU	PV	1862	D4: ~7 × 10^5^	D4: < LLOD	(72)
BNT162b2	100 μg	2	3	1.05 × 10^6^ PFU	PV	310	D3: ~1 × 10^6^	D4: < LLOD	(73)
Ad26.COV2.S	5 × 10^10^ vp	2	8	1 × 10^5^ TCID50	PV	~1000	D4: ~1 × 10^5^	D4: < LLOD	(74)
ChAdOx1 nCoV19	2.5 × 10^10^ vp	1	_	2.6 × 10^6^ TCID50	Live-CPE	~20 (5–40)	D3: ~1 × 10^5^	D3: < LLOD	(75)
	2.5 × 10^10^ vp	2	4	2.6 × 10^6^ TCID50	Live-CPE	10–160	_	_	(76)
BBIBP-CorV	2/8 μg	2	2	10^6^ TCID50	Live-CPE	215/256	~1× 10^3^-1 × 10^6^ (lung)	< LLOD (lung)	(77)
PiCoVacc	6 μg	3	1	10^6^ TCID50	Live-CPE	~50	~1× 10^3^-1 × 10^6^ (lung)	< LLOD (lung)	(78)
BBV152	3 μg	2	2	1.25 × 10^6.5^ TCID50	Live-PRNT	~3100	D3: ~1 × 10^6^	< LLOD	(79)
INO-4800	1 mg	1/2	4	5 × 10^6^ PFU	Live-PRNT	2199	1 × 10^6^	1 × 10^4^	(80)
NVX-Cov2373	50 μg	2	3	1.1 × 10^4^ PFU	Live-CPE	23040	sgRNA ~ < 1E4	< LLOD	(81)
SCB-2019	30 μg	2	3	2.6 × 10^6^ TCID50	Live-CPE	2700/35047	D2: 1 × 10^4^	< LLOD	(82)

vp, viral particles; PFU, plaque-forming units; TCID_50_, tissue culture infective dose 50; CPE, cytophatic effect detection assay; PRNT, plaque reduction neutralisation test; LLOD, lower limits of detection; BAL, Bronchoalveolar lavage.

### Phase III Clinical Trials Data

In recent months, vaccine manufacturers have published phase III COVID-19 vaccine clinical trial data, which have indicated vaccine efficacies consistent with those reported in phase I/II clinical trials. Although differences exist among specific vaccines, the various vaccines have exhibited good effects in the prevention of disease onset and critical illness (**Table 3**). At present, data related to the correlation between efficacy and neutralizing antibody titer in phase III clinical trials have not yet been reported. A meta-analysis that compared the correlations between efficacy and neutralization titer in recovering subjects across several phase III clinical trials revealed the presence of a strong nonlinear relationship between mean neutralization level and efficacy, which is in agreement with results obtained from animal challenge models (28). However, inconsistencies in the selected serum samples of the recovery phase may reduce the credibility of this relationship. In addition, large differences exist in the neutralizing antibody titers induced by similarly efficacious vaccines developed by different manufacturers. This may be attributed to differences in the sample population, test methods, tested variants, and dominant variant in the country of residence of the subjects, among phase III clinical trials conducted by different vaccine manufacturers (**Table 3**). In a recent study, Feng et al. analyzed the clinical data of the ChAdOx1 nCoV-19 (AZD1222) vaccine in the UK and found that the vaccine efficacy was associated with antibody levels (especially those of neutralizing antibodies). Measurements of neutralizing antibody titers revealed values of 938 international units (IU)/mL was associated with 90% VE against symptomatic infection at 28 days post-vaccination (71). A study on breakthrough infections in vaccinated healthcare workers at the largest medical center in Israel predicted that breakthrough infections could only be effectively prevented when the geometric mean titer (GMT) exceeded 533.7 (95% CI: 408.1 to 698.0) (98).

**Table 3:** Neutralizing antibody titer and protection efficacy of COVID-19 vaccines for emergency use in Phase III clinical trials.

Vaccine	Clinical trial No.	Country	No. of participants	Age	Efficacy (%) (95% CI)	Neutralizing antibodies titer to live SARS-CoV-2	Ref
						Geometric mean ratio (95% CI) (SARS-CoV-2 strain)	
BBIBP-CorV	NCT04510207	UAE, Bahrain	40382	≥ 18	78.1 (64.8–86.3)	68.7 (65.5–72.1) [19nCoVCDC-Tan-Strain04 (QD01)]	(83)
Inactivated (Wuhan, Sinopharm)	NCT04510207	UAE, Bahrain	40382	≥ 18	72.8 (58.1–82.4)	41.0 (38.9–43.2) (19nCoVCDC-Tan-Strain04 (QD01))	(83)
CoronaVac	NCT04456595	Brazil	9823	≥ 18	50.7 (36.0–62.0)	64.4 (B.1.128 (SARS-CoV-2/human/(BRA/SP02/2020 strain MT126808.1)	(84)
						46.8 (SARS-CoV-2-P.1 MAN 87201 strain)	
						45.8 (SARS-CoV-2-P.2 LMM38019 strain)	
CoronaVac	NCT04582344	Turkey	10214	18–59	83.5 (65.4–92.1)	–	(85)
ChAdOx1(AZD1222)	NCT04324606 NCT04400838 NCT04444674 ISRCTN89951424	UK, Brazil, South Africa	11636	≥ 18	62.1 (41.0–75.7)	51 (32–103)*	(86, 87)
Sputnik V	NCT04530396	Russia	21977	≥ 18	91.6 (85.6–95.2)	44.5 (31.8–62.2) (hCoV-19/Russia/Moscow_PMVL-1/2020)	(88)
BBV152	NCT04641481	India	25798	18–98	77.8 (65.2–86.4)	125.6 (111.2-141.8)	(89)
mRNA-1273	NCT04470427	United States	30420	≥ 18	94.1 (89.3–96.8)	654.3(460.1–930.5)*	(90, 91)
BNT162b2	NCT04368728	United States, Argentina, Brazil, South Africa Germany, Turkey	43448	≥ 16	95.0 (90.3–97.6).	363*	(92, 93)
Ad26.COV2.S	NCT04505722	Argentina, Brazil, Chile, Colombia, Mexico, Peru, South Africa, United States	39321	≥ 18	66.9 (59.0–73.4)	827 (508–1183)* (Victoria/1/2020 SARSCoV-2 strain)	(94, 95)
NVX-CoV2373	EudraCT number, 2020-004123-16	United Kingdom	14039	18–84	89.7 (80.2–94.6)	3906*	(96, 97)

*Represents that these data were reported in phase I or II clinical trials.

### Real-World Data

With the publication of real-world data, it is apparent that current COVID-19 vaccines that have been granted EUAs or conditional marketing authorizations, achieve different efficacies in individuals of different age groups, ethnicities, and countries. However, all vaccines have met the minimum efficacy requirement of 50% set by the WHO for EUAs and demonstrated high efficacies against progression towards severe disease or death (**Table 4**) (26, 99–102). The effectiveness of the vaccines is positively correlated with the level of neutralizing antibodies (**Table 3**), which is consistent with Knory’s and Earle’s studies (28, 29). Although the efficacies of current vaccines against the SARS−CoV−2 variants of concern (VOC) have decreased significantly compared to the preclinical stage, these vaccines still provide certain protective effects, especially against critical illness and disease-related death (27, 102, 103).

**Table 4:** Efficacy of COVID-19 vaccines in real word.

Vaccine	Country	No. of participants	Age	Efficacy (%) (95%CI)				Ref
				prevention of Covid-19	prevention of hospital	prevention of severe disease	prevention of death	
CoronaVac	Chile	10,187,720	≥16	65.9 (65.2- 66.6)	87.5 (86.7-88.2)	90.3 (89.1-91.4)	86.3 (84.5- 87.9)	(26)
BNT162b2, mRNA-1273	Canada	324 033	≥16	91 (89- 93)	98 (88- 100) (hospital or death)			(99)
BNT162b2	Israel	119,236	≥16	92 (88- 95)	87 (55-100)	92 (75- 100)	–	(100)
BNT162b2	Qatar	–	–	–	–	97.4 (92.2- 99.5)		(101)
BNT162b2*	Unite States	51,738	≥18	76 (69-81)	85 (73-93)	–	–	(102)
mRNA-1273*	Unite States	51,738	≥18	86 (81-90.6)	91. (81-97)	–	–	(102)

*Alpha or Delta variant was highly prevalent in this region in this study.

The decrease in the efficacy of current vaccines has mainly been caused by the following: (1) Neutralizing antibodies generated after vaccination with current vaccines providing weaker neutralizing effects against newly emerged variants, resulting in decreased vaccine efficacy (104, 105); (2) Neutralizing antibody levels starting to decrease gradually at a certain point of time after vaccination, resulting in the occurrence of breakthrough infection. Consequently, populations that have been vaccinated earlier are more susceptible to breakthrough infection than those vaccinated later (106–108). Studies have found that booster vaccination leads to increased neutralizing antibody levels against variants and enhances vaccine efficacy (49, 50, 52). Both clinical and real-world data demonstrate the presence of a positive correlation between neutralizing antibody level and vaccine effectiveness, which provides a scientific basis for further data collection and analysis, to validate the use of neutralizing antibody threshold values as surrogate endpoints.

### Application of Neutralizing Antibody Immunobridging for COVID-19 Vaccine

In recent clinical studies of two COVID-19 vaccines that were performed around the world, neutralizing antibody immune bridging was used to assess vaccine’s effectiveness. The two co-primary endpoint criteria used were: 1. The neutralizing antibody titer is higher compared to active comparator vaccine AZD1222 (ChAdOx1-S); 2. The seroconversion rate of neutralizing antibody is more than the set threshold of 50% or 95% (109, 110). After full immunization schedule, seroconversion of SARS-CoV-2-specific neutralizing antibodies is defined as 4-fold increase from baseline in the phase III clinical of VLA2011 (111). The Phase II clinical results of MVC-COV1901 showed that the geometric mean serum neutralizing antibody titer at 28 days after receiving the second recommended dose (i.e. Day 57) against wild type SARS-CoV-2 virus was 408.5 IU/mL. The neutralizing antibody GMT induced by MVC-COV1901 was 3.4 times that of AZD1222, and the seroconversion rate was 99.8% (109, 112–114). On July 19, 2021, without providing clinical research data on vaccine protection, MVC-COV1901 was approved by the EUA of Taiwan, China, becoming the first approved COVID-19 vaccine based on neutralizing antibody bridging experiments to evaluate immune protection (109, 114). Phase III clinical results of the AZD1222 vaccine showed an efficacy of 62.1%. Based on the study of the relationship between the level of neutralizing antibody and vaccine immunity by Feng et al., it can be inferred that the protection rate of the MVC-COV1901 vaccine is between 80% and 90% (71, 86). On October 18, 2021, Valneva reported positive phase 3 results for VLA2001. The neutralizing antibody titer at two weeks after the second receiving the second recommended dose (i.e. Day 43) in adults aged 30 years and older of VLA2001 is 1.39 times that of AZD1222 (VLA2001 GMT 803.5, AZD1222 GMT 576.6), and the neutralizing antibody seroconversion rate is more than 95% (110, 115). The efficacies of MVC-COV1901 and VLA2001 vaccines will be evaluated based on real-world vaccination data. This will be the direct and effective verification of the feasibility of using neutralizing antibodies as surrogate endpoints for COVID-19 vaccines.

### Neutralizing Antibody Test Methods

Neutralizing antibody testing can be performed using live virus, pseudovirus neutralization assays and lateral flow immunoassay (116). A report by the WHO revealed the presence of differences in the experimental methods, variants, and calculation methods used for neutralizing antibody testing among different laboratories around the world, which resulted in significant biases in the measured neutralizing antibody levels of the same sample. In the collaborative calibration of the First WHO International Standard and Reference Panel for the anti-SARS-CoV-2 antibody, live virus neutralization assays performed by 15 laboratories [including live virus plaque reduction neutralisation assay (Live-PRNT), live virus foci reduction neutralisation assay (Live-FRNT), live virus cytophatic effect detection assay (Live-CPE), and live virus microneutralization assay (Live-MN)] and pseudovirus neutralization assays performed by 12 laboratories [including pseudotyped virus - lentiviral (HIV) vector (PV-LVV) and pseudotyped virus - vesicular stomatitis virus (PV-VSV)] were adopted (117). Results indicated that with the exception of two low-titer samples and one negative sample for which the titers could not be easily calculated, the total GMTs of seven collaborative calibration samples determined by Live-PRNT, Live-FRNT, Live-CPE, and Live-MN were 317.1, 445.3, 93.9, and 239.6, respectively. When the same assay was used, the average fold (the ratio of the maximum value to the minimum value) in GMT across different laboratories were 14.7-, 12.9-, 19.5-, and 4.5-fold, respectively. Total GMTs determined by PV-LVV and PV-VSV were 371.8 and 519.2, respectively; average fold in GMT across different laboratories when the same method was used were 908.7- and 10.9-fold, respectively.

During the collaborative calibration of the first Chinese national standards for SARS-CoV-2 neutralizing antibody, live virus neutralization assays performed by four laboratories (including Live-PRNT and Live-CPE), with one laboratory adopting two types of assays for testing and the remaining three laboratories using the CPE assay, and pseudovirus neutralization assays performed by 10 laboratories (all PV-VSV) were adopted. As shown in **Table 5**, the GMTs of four collaborative calibration samples determined by different assays are significantly inconsistent. When the same assay was used, the fold (the ratio of the maximum value to the minimum value) in GMT across different laboratories are more than 4.7 at least. These results indicate the presence of considerable differences among different laboratories and methods (**Table 5**) (118).

**Table 5:** Comparison of geometric mean of SARS-CoV-2 neutralising antibodies reported in ref. (118).

Type	No. of lab	Sample							
		22		44		77		99	
		GMT	Fold	GMT	Fold	GMT	Fold	GMT	Fold
Live-CPE	4	176.6	36.9	728.2	6.0	38.7	10.7	469.4	7.6
Live-PRNT	1	1063	–	2308	–	183	–	1463	–
PV-VSV	10	1938	4.8	3973	4.7	162	23.1	2064	10.5

Fold, the ratio of the maximum value to the minimum value; FRNT, foci reduction neutralization assay; PV, pseudotyped virusbased neutralization assay; VSV, vesicular stomatitis virus.

Studies have shown that differences in neutralizing antibody test results among different laboratories were significantly decreased with the use of the WHO International Standard and the Chinese National Standards (117, 118). When the International Standard was adopted, the total geometric coefficients of variation (GCVs) of five high- and medium-titer samples were reduced from 249%, 179%, 231%, 281%, and 161% to 94%, 95%, 119%, 67%, and 93%, and the upper quartile/lower quartile (UQ/LQ) values were reduced by 1.432-, 1.978-, 2.206-, 6.511-, and 2.348-fold. However, a low-titer sample did not exhibit a significant decrease in GCV and only showed a 0.97-fold decrease in UQ/LQ compared with the pre-standardization value, which may be related to the fact that the low neutralizing antibody titer was close to the threshold value (117). In the collaborative calibration of the Chinese national standards, the GCV values of three collaborative calibration samples measured by different laboratories using an authentic virus neutralization assay were 129%, 266%, and 146%. When standards with a titer of 1000 U/mL were used, the total GCVs among laboratories were reduced to 107%, 18%, and 90% (118).

The standardization of neutralizing antibody test methods directly affects the establishment of immunological surrogate endpoints and has become a major influencing factor of COVID-19 vaccine R&D and evaluation. With the establishment of the first standard pseudovirus neutralization assay by Chinese researchers (119) and WHO’s subsequent approval of the First International Standard for anti-SARS-CoV-2 immunoglobulin (human), methods for measuring neutralizing antibody titer can be standardized and the comparability of cross-platform test results can be effectively enhanced (120).

## Actions to Develop Neutralizing Antibodies as Endpoint for Vaccines of WHO and More National Levels of Different Countries to Practical Application

As trials on the clinical effectiveness of vaccines constitute the main rate-limiting step in vaccine R&D, the current status of COVID-19 vaccine application and R&D urgently require the establishment of immunogenicity surrogate endpoints for testing vaccine’s effectiveness. Recently, a number of reports on clinical and real-word effectiveness of COVID-19 vaccines have been published, and two COVID19 vaccines have reported positive comparative immunogenicity trial results (109–114). We are beginning to harness the potential utility of these data for establishing surrogate endpoints. However, the current guidance documents on immunological surrogate endpoints are not systematic and comprehensive. There are obstacles to sharing and analyzing large amounts of clinical data among vaccines manufactures. In addition, the lack of a standardized neutralizing antibody test assay and continuously emerging variants further complicate the comparisons. The robustness of immunological surrogate endpoints will largely depend on how we address these issues.

### Guidance Documents on Surrogate Endpoints for COVID-19 Vaccines Promptly Issued by the WHO and National Regulatory Agencies

The pandemic of COVID-19 requires the WHO and national regulatory agencies to quickly assess the protective effectiveness of vaccines. Recently, the WHO and pharmaceutical regulatory agencies of the USA, UK, and China have indicated the need for studies investigating the relationships between vaccine-induced neutralizing antibody levels and vaccine’s effectiveness (**Table 6**). In particular, the Medicines and Healthcare products Regulatory Agency (MHRA) of the UK has announced that neutralizing antibody surrogate endpoints established using the WHO standard units can be applied to immunobridging studies in the R&D of vaccines against SARS−CoV−2 variants (121–124).

**Table 6:** Guidance for COVID-19 vaccine R&D.

Regulatory agency	Guidance document	Date of issue	Requirements for surrogate endpoints	Ref
WHO	Guidance on conducting vaccine effectiveness evaluations in the setting of new SARS-CoV-2 variants: Interim guidance	2021.07.22	An approach to better estimate the vaccine effectiveness for new variants is looking for concordance of neutralization data and vaccine effectiveness results for new variants, which would add credibility to the vaccine effectiveness estimate.	(121)
EMA	Reflection paper on the regulatory requirements for vaccines intended to provide protection against variant strain(s) of SARS-CoV-2	2021.02.23	In the absence of an immune correlate of protection, the evaluation of the neutralizing antibody levels elicited by the vaccines against variants and the parent strain under standardized test conditions is required to serve as a secondary endpoint.	(122)
CDE (China)	Technical guidelines for the development of novel coronavirus preventive vaccines (trial)	2020.08.14	The investigation of correlations between immunogenicity markers and protection in the evaluation of clinical efficacies of vaccines is recommended. Surrogate endpoints should be explored, and the investigation of correlations between vaccine immunogenicity and effectiveness, and reasonable immunological surrogate endpoints are encouraged during clinical R&D of vaccines. The use of neutralizing antibody levels as surrogate endpoints requires evidence in the following five aspects: (1) Viral pathogenesis and mechanisms underlying immune response to the virus; (2) Relationships of viral infection-induced serum antibody levels with disease onset, progression, and outcome; (3) Relationship of the serum antibody level with vaccine efficacy and the predicted values; (4) Immune response after vaccination, production/non-production of neutralizing antibodies, and neutralizing antibody levels; (5) Neutralizing antibody levels during the effective period of protection and correlation with vaccine efficacy.	(123)
FDA (USA)	Emergency use authorization for vaccines to prevent COVID-19; Guidance for industry	2021.05.25	When evaluating vaccines targeted against new variants, the neutralizing antibody may be considered a relevant measure of immunogenicity. Data demonstrating the ability of new COVID-19 vaccines to induce a neutralizing antibody response are needed, which may be derived by assessing the neutralization of SARS-CoV-2 viruses (including the virus from which the prototype vaccine was derived as well as variants of interest) with clinical serology samples.	(124)
MHRA (UK)	Decision: Access consortium: Alignment with ICMRA consensus on immunobridging for authorizing new COVID-19 vaccines	2021.09.15	Based on the specifics of the product under consideration, a neutralizing antibody titer may be justified as an immune marker to predict vaccine effectiveness. However, neutralizing antibody titers should be determined using the WHO-certified reference standards. The weight of evidence from studies with authorized COVID-19 vaccines is sufficient to support the use of a neutralizing antibody titer as a primary endpoint in cross-platform immunobridging trials.	(125)

Guidelines issued by the Center for Drug Evaluation (CDE) of the National Medical Products Administration (NMPA) of China state that the correlation between immunological markers and protection should be investigated in COVID-19 vaccine clinical trials, and the establishment of surrogate endpoints requires the provision of evidence for five different aspects, which include the correlations of immune response mechanisms and antibody levels (particularly those of neutralizing antibodies) with protection (123). Considering the fact that the current global outbreaks are caused by SARS−CoV−2 variants, the Unite States Food and Drug Administration (USFDA) has indicated the need to assess the neutralization of the virus from which the prototype vaccine was derived, as well as variants of concern with clinical serology samples obtained from persons immunized with vaccines against variants, when investigating the effectiveness of newly developed vaccines against variants (124). Guidance provided by the WHO recommends the approach of looking for concordance of neutralization data and vaccine effectiveness results for VOCs to estimate the effectiveness of vaccines against new variants (121). The MHRA considers that the weight of evidence from studies with authorized vaccines is sufficient to support the use of neutralizing antibody titers as a primary endpoint in cross-platform immunobridging trials. Therefore, neutralizing antibody titers may be justified as an immune marker to predict vaccine effectiveness, but they should be standardized using the WHO reference standards and expressed in terms of IU (125). To guide deliberations regarding vaccines against variants, the European Medicines Agency (EMA) requires the evaluation of the neutralizing antibody levels elicited by the vaccines against variants and the parent strain under standardized conditions to serve as a secondary endpoint (122).

### Analysis of the Use of Phase III Clinical and Real-World Data as Surrogate Endpoints

Currently, 31 different types of COVID-19 vaccines have received EUAs or conditional marketing authorizations, and preclinical, clinical, and real-world data on efficacy and neutralizing antibody levels are available for many of these vaccines. However, inadequacies exist in the openness and correlation analyses of the efficacy and neutralizing antibody data of current vaccines (28, 71).The clinical studies of MVC-COV1901 and VLA2001 vaccines provide the results of immune bridging of neutralizing antibodies, but there is a lack of data on the protection rate of vaccines. These data could be jointly analyzed by vaccine manufacturers and regulatory agencies, and coordination efforts could be made by international health organizations for the standardized formulation of scientific surrogate endpoints. These will be greatly beneficial to the screening of high-immunogenicity vaccines from the immense number of vaccines being subjected to preclinical and clinical testing worldwide, maximization of the protection of participants and saving various resources. On the basis of existing clinical data, statistical tools may be utilized to investigate the relationships between neutralizing antibody level and vaccine efficacy, changes in neutralizing antibody level with time, and threshold levels of neutralizing antibody protection against different variants (28, 71). However, neutralizing antibodies are likely not the only mechanism of protection (126). Further clinical and real-world data are required to determine the ability of neutralizing antibody levels to accurately evaluate the effectiveness of COVID-19 vaccines when used as surrogate endpoints.

### Standardization of Laboratory Serological Test Methods and the Establishment of Secondary National Standards

There are a number of neutralizing antibody test methods available for the estimation of antibodies against SARS-CoV2 (116). Standardized laboratory serological test methods are a prerequisite for the realization of data comparability among different laboratories and platforms for surrogate endpoint establishment. The standardization of test methods has become the main rate-limiting step in the R&D of COVID-19 vaccines. On the basis of the WHO antibody standard, countries and regions are advised to expedite the establishment of secondary national standards to specify requirements for the use of IU in test methods. This will enable the comparison of neutralizing antibody test results across different studies for the accurate analysis and determination of correlations between neutralizing antibody levels and vaccine efficiencies.

### New Variants Pose Challenges to the Establishment of Surrogate Endpoints

SARS−CoV−2 is highly prone to mutations. To date, several hundreds of lineages have already emerged and new lineages are still appearing on a continuous basis. In particular, lineages with immune evasion abilities exert greater effects on vaccine protection (127). Serum neutralizing capacity has been used as an efficient indicator to quickly assess the protective effect of vaccines on emerging variants. Results of these studies show that compared with the early strains, the neutralizing antibody titer against new variants has been significantly decreased (5, 128, 129). Surrogate endpoints established using current clinical data will inevitably face challenges posed by continuously emerging variants. Therefore, continuous endpoint revisions may be required based on changes in variant dominance with time and the R&D status of vaccines (130).

In addition, SARS-CoV-2 specific T cell immunity acquired by COVID-19 vaccines or previous infection still remain broadly robust and long-term protection against VOCs, including Omicron variant (131–134). A standardized measurement of T cells immunity may be served as an potential surrogate endpoint to better assess the protective effect of COVID-19 vaccines on emerging variants subsequently (135).

## Conclusion

The global scale of the pandemic, high vaccination coverage rates and ethical requirements, pose challenges to the effectiveness of subsequently developed COVID-19 vaccines in clinical trials. The establishment of immunological surrogate endpoints is of great significance to the acceleration of efficacy evaluations of vaccines. Current studies have indicated that vaccine-induced neutralizing antibody levels are correlated with clinical protection, and predictable clinical progression have tried to assess clinical protection through neutralizing antibody immunobridging experiments (109, 110). Research efforts on surrogate endpoints should focus on the establishment and application of standardized test methods, and adoption of the WHO international standard to express titers in terms of IU and reduce measurement errors. In addition, it should also analyze the threshold levels of neutralization antibody protection against different variants based on current clinical data, and appropriate endpoint adjustments based on changes in variant circulation and vaccine R&D. Due to the rapid waning of neutralization tier, it is critial to assess quality and durability of the neutralization antibody in conjunction with standardization of time lines after vaccination (28). During the use of COVID-19 vaccines in clinical trials of booster and sequential vaccination, the threshold levels of new vaccines’ neutralization antibody should be significantly superior to those of the primary vaccines. Higher titers of neutralization antibody will serve as an indication of the superior effectiveness of these new vaccines.

## Author Contributions

ZL and MX conceived the framework and main text of this review article. JL, QM, XW, QH, and LB wrote the draft. ZL, MX, ZW and QW reviewed the manuscript. JL, YB, and JZ searched the literature. All authors contributed to the article and approved the submitted version.

## Funding

This work was supported by the Emergency Key Program (NO. EKPG21-30-1) of Guangzhou Laboratory, China.

## Conflict of Interest

The authors declare that the research was conducted in the absence of any commercial or financial relationships that could be construed as a potential conflict of interest.

## Publisher’s Note

All claims expressed in this article are solely those of the authors and do not necessarily represent those of their affiliated organizations, or those of the publisher, the editors and the reviewers. Any product that may be evaluated in this article, or claim that may be made by its manufacturer, is not guaranteed or endorsed by the publisher.
